# Lowering Nitrogen and Increasing Potassium Application Level Can Improve the Yield and Quality of *Panax notoginseng*

**DOI:** 10.3389/fpls.2020.595095

**Published:** 2020-12-21

**Authors:** Xiaohong Ou, Xiuming Cui, Duanwei Zhu, Lanping Guo, Dahui Liu, Ye Yang

**Affiliations:** ^1^Yunnan Provincial Key Laboratory of Panax notoginseng, Key Laboratory of Panax notoginseng Resources Sustainable Development and Utilization of State Administration of Traditional Chinese Medicine, Kunming Key Laboratory of Sustainable Development and Utilization of Famous-Region Drug, Faculty of Life Science and Technology, Kunming University of Science and Technology, Kunming, China; ^2^Resource Institute for Chinese Medicine and Ethnic Materia Medica, Guizhou University of Traditional Chinese Medicine, Guiyang, China; ^3^College of Environment and Resources, Huazhong Agricultural University, Wuhan, China; ^4^Chinese Medica Resources Center, China Academy of Chinese Medicinal Sciences, Beijing, China; ^5^College of Pharmacy, Hubei University of Traditional Chinese Medicine, Wuhan, China

**Keywords:** *Panax notoginseng*, nitrogen and potassium, balanced fertilization, yield, saponins, photosynthesis

## Abstract

Excessive nitrogen (N) application and potassium (K) supplement deficiency is a common problem in *Panax notoginseng* cultivation. However, synergistic effects of lowering N and increasing K application on yield and quality of *P. notoginseng* have not been reported. Field experiments in two locations with different N and K combined application were conducted to study the effects on yield and quality. Then, the saponin accumulation mechanisms were explored by pot and hydroponic culture with 2- or 3-year-old seedlings. The investigation showed that 70% of *P. notoginseng* cultivation fields reached abundant levels of total nitrogen (TN) but had deficient levels of total potassium (TK), which may be detrimental to balance the N/K uptake of *P. notoginseng*. Moreover, the average biomass was 18.9 g, and *P. notoginseng* saponin (PNS) content was 6.95%; both were influenced by the N/K values of *P. notoginseng*. The field experiments indicated that compared to the conventional N and K application (N:K = 2:1), lowering N and increasing K application (N:K = 1:2) decreased root rot rate by 36.4–46.1% and increased survival rate, root biomass, and yield, as well as PNS content by 17.9–18.3, 5.7–32.9, 27.8–57.1, and 5–10%, respectively. The mechanism of lowering N and increasing K application on the PNS content improving was due to the decreasing of N/K value, which promoted photosynthesis, sugar accumulation, and the expression of saponin biosynthesis genes. Therefore, lowering N and increasing K application to the ratio of 1:2 would have great potential to improve the synergistic effect on yield and quality of *P. notoginseng* cultivation.

## Introduction

*Panax notoginseng* (Burk.) F. H. Chen (*P. notoginseng*), belonging to the *Araliaceae* family, a famous traditional Chinese medicine for the prevention and treatment of cardiovascular and cerebrovascular diseases (Ng, 2006), has been planted for more than 400 years (Wan et al., 2012). Until now, more than 100 kinds of saponins have been identified (Wang et al., 2016), of which notoginsenoside R_1_ and ginsenoside R_g__1_, R_b__1_, R_e_, and R_d_ are the five most common kinds. Many well-known traditional Chinese patent medicines used its dry root as the main raw material, such as Yunnan Baiyao, Xuesaitong. Moreover, the revenue from cultivation, processing, and other related industries of *P. notoginseng* has become one of the most important economic pillars of Yunnan province in China.

With the increasing of market demand, the raw material price of *P. notoginseng* has risen to a very high level. For this reason, farmers always excessively fertilize for maximum yield. A survey showed that the N application rate in conventional cultivation of *P. notoginseng* is 225–450 kg.hm^–2^ (Xia et al., 2016), which not only exceeded its N demand (Ou et al., 2011), but also exacerbated its disease incidence (Wei et al., 2018). Thus, research suggests the rational N application rate for *P. notoginseng* cultivation should be less than 225 kg.hm^–2^ (Wei et al., 2018). Moreover, excessive N application could decrease the K use efficiency (Peng et al., 2006; Zhang et al., 2010; White, 2013), resulting in a relative K deficiency, as well as attenuating K fixation potential of the planting soil (Kolahchi and Jalali, 2007). Recently, Du et al. (2016) investigated 57 sites, which adopt the conventional fertilization method for *P. notoginseng* cultivation, and found more than 76% of the cultivation soils reached excessive levels of available potassium (AK), whereas about 97% of them had deficient levels of total potassium (TK). These findings suggest that *P. notoginseng* cultivation soil has a common problem of low K fixation and supplying potential. In addition, our previous field survey found the practical K application rate was about 150–225 kg.hm^–2^ (Hao et al., 2014), which might not be sufficient for root yield improving of *P. notoginseng*. Many studies showed that increasing K application could enhance the disease resistance of crops (Amtmann et al., 2008; Oosterhuis et al., 2013; Wang et al., 2013; Zörb et al., 2014). Studies of our group have demonstrated the demand of *P. notoginseng* for macroelements follows an order of K > N > P (phosphorus) (Ou et al., 2011), and the recommendation application rate of K_2_O should be more than 300 kg.hm^–2^ (Song et al., 2019). But, the current customary application amount of N is more than 225 kg.hm^–2^, and K application is less than 225 kg.hm^–2^ in *P. notoginseng* cultivation. Thus, the synergistic effect of rational combined application of N and K on the disease incidence, yield, and quality has not been conducted in *P. notoginseng* cultivation until now.

Therefore, in the present study, in order to lay out the potential of lowering N and increasing K application amount in improving efficiency of *P. notoginseng* cultivation, field investigation and designed experiments were performed. The correlation of soil N and K contents with biomass and quality of *P. notoginseng* were fitted using field investigation data. Subsequently, 2-year field and pot culture experiments were conducted to further verify the promoting effects of a balanced N/K on the growth, quality, and photosynthesis of *P. notoginseng*. In addition, a hydroponic experiment was designed to elucidate the biological mechanism of balanced application of N and K and enhance *P. notoginseng* saponin (PNS) accumulation in *P. notoginseng*.

## Materials and Methods

### Field Investigation

During year 2013–2014, 33 *P. notoginseng* cultivation fields were investigated. From each field, a soil sample of 0–20 cm was collected using a “five-point” method; a plant sample with 20 3-year-old *P. notoginseng* seedlings was randomly sampled. All soil samples were air dried and smashed for the measuring of N and K content, and all plant samples were divided into leaf and root, aired, and smashed for the measuring of N, K, and PNS content.

### Field Experiment

The field experiment was repeated two times at two experiment sites from year 2016 to 2019. The experiment of year 2016–2017 was conducted at Xiaoyize village, Shizong county, Yunnan province, China (E:103°55′52″; N:24°44′40″), and the experiment of year 2018–2019 was conducted at Malishu village, Qiubei county, Yunnan province, China (E: 104°6′59″; N:23°59′3″). The previous crops of the both fields were corn; soil characteristics are shown in Table 1. The field experiments were conducted by random block design with a plot size of 2.3 × 1.9 m. According to the levels of conventional N and K application, as well as the recommendation application levels of N 150 kg.hm^–2^ (Ou et al., 2011) and K_2_O 300 kg.hm^–2^ (Song et al., 2019), this study conducted four N and K combination treatments with four replications, referred to as N1K1, N1K2, N2K1, and N2K2. N2 was the conventional N application treatment; N1 was the N-lowering application treatment (recommendation). K1 was the conventional K application treatment; K2 was the K-increasing application treatment (recommendation). Urea (46%, N), calcium magnesium phosphate fertilizer (P_2_O_5_, 12%), and potassium sulfate (K_2_O, 50%) were, respectively, used for N, P_2_O_5_, and K_2_O supplements, and the application amount and application methods are shown in Table 2. Except the N and K application ways, the other management was the same with the local practices, and the following were carried out: seedlings of 1 year old (bought from Wenshan Sanqi trading market) were transplanted, with a density of 15 × 15 cm in December of the first year; the roots (called 3-year-old *P. notoginseng*) were harvested in November of the third year.

**TABLE 1:** Soil characteristics for the field and pot culture experiments.

**Soil traits**	**Unit**	**Field experiments**		**Pot culture**
		**Shizong (2016–2017)**	**Qiubei (2018–2019)**	
Soil type	—	Red clay		
pH	—	5.57	6.20	7.32
Organic matter (OM)	g.kg^–1^	5.06	8.20	11.76
Total nitrogen (TN)	g.kg^–1^	1.06	0.82	0.67
Total phosphorus (TP)	g.kg^–1^	0.49	0.80	0.57
Total potassium (TK)	g.kg^–1^	4.10	11.30	3.56
Available nitrogen (AN)	mg.kg^–1^	61.00	85.70	48.22
Available phosphorus (AP)	mg.kg^–1^	1.20	58.30	6.78
Available potassium (AK)	mg.kg^–1^	72.00	176.50	113.39

**TABLE 2:** The fertilization procedure of different treatments in the field experiment.

**Index**		**N1K1**	**N1K2**	**N2K1**	**N2K2**	**Fertilization procedure**
First year (kg.hm^–2^)	P_2_O_5_	150				Base (100%, 1-time)
	N	150	150	300	300	Base (30%): Topdressing [70%, 4-time, 20% (May)–10% (Jun.)–20% (Aug.)–20% (Oct.)]
	K_2_O	150	300	150	300	Base (30%): Topdressing [70%, 4-time, 20% (May)–10% (Jun.)–20% (Aug.)–20% (Oct.)]
Second year (kg.hm^–2^)	P_2_O_5_	225				Topdressing [2-time, 50% (Mar.)–50% (Jul.)]
	N	225	225	450	450	Topdressing [5-time, 20% (Mar.)–30% (May)–10% (Jun.)–20% (Aug.)–20% (Oct.)]
	K_2_O	225	450	225	450	Topdressing [5-time, 20% (Mar.)–30% (May)–10% (Jun.)–20% (Aug.)–20% (Oct.)]

### Pot Experiment

The pot experiment was conducted from May to November 2017 at the experimental station of Kunming University of Science and Technology, located at Kunming, Yunnan, China (E:102°51′49.85″; N:24°50′39.69″). Soil was taken from the station, air dried, removed dopant, smashed, sieved, and soil characteristics, shown in Table 1. Plastic pots (size: 70 × 40 × 28 cm) were filled with 18-kg soil for the pot culture experiment. Two- or 3-year-old seedlings bought from Wenshan Sanqi trading market were transplanted. According to the application levels of the field experiments in 20-cm-deep tillage soil, the N application levels were converted and adjusted. The conventional and lowered N application rates were 0.5 and 0.3 g.kg^–1^ soil, respectively; the conventional and increased K application rates were 0.3 and 0.5 g.kg^–1^ soil, respectively. Therefore, four treatments with five replicates were designed and referred to as N_0_._3_K_0_._3_, N_0_._3_K_0_._5_, N_0_._5_K_0_._3_, and N_0_._5_K_0_._5_. The P_2_O_5_ application rate of each treatment was 0.3 g.kg^–1^ soil. To ensure that the microelements were sufficient for normal growth, 5 mL of microelements solution was added to every 1 kg soil, referred to Ou et al. (2014). The components of the solution were 46 μM H_3_BO_3_, 9 μM MnSO_4_, 10 μM Na_2_EDTA-FeSO_4_, 0.8 μM ZnCl_2_, 3.2 μM CuSO_4_, and 0.1 μM Na_2_MoO_4_. The fertilization method followed the procedure with total P_2_O_5_, K_2_O, 50% of total N as basal, and 50% of total N as topdressing. Basal fertilizers were mixed with soil of each pot. Topdressing N was equally applied in the second and third month after transplantation. All the four treatments were divided into two groups, one was transplanted 2-year seedlings (eight plants per pot) and one was transplanted 3-year seedlings (six plants per pot). Before transplantation, the soil on the roots of each seedling was removed and then weighted. Other management procedures were the same as the field management.

### Hydroponic Experiment

According to our previous study, the 3-year *P. notoginseng* seedlings can grow normally in 5 mM ammonium solution, but were inhibited in 15 mM ammonium (Ou et al., 2019). Thus, the concentration of N in the hydroponic was adopted 5 mM. Then, three K concentrations with three biological replications, 5, 10, and 15 mM, were selected, and were signed as N5K5, N5K10, and N5K15, respectively. The N and K in nutrient solution were supplied by (NH_4_)_2_SO_4_ and K_2_SO_4_. Other nutrients in each solution were the same as follows: 3 mM KH_2_PO_4_, 2.5 mM CaSO_4_, 1 mM MgSO_4_, 50 μM Na_2_EDTA-FeSO_4_, 7 μM MnSO_4_, 0.7 μM ZnCl_2_, 0.8 μM CuSO_4_, 2 μM H_3_BO_3_, and 0.8 μM Na_2_MoO_4_, and the solution pH value was adjusted to 6.0–6.1. In this experiment, in total, 54 3-year-old seedlings were washed clean and pretreated with 0.01 M CaCl_2_ for 24 h, and then each replication transplanted six seedlings. After 24-h treatment, the seedling root in each replication was separately washed and then mixed, frozen with liquid nitrogen, and stored at −80°C for RNA isolation and real-time quantitative polymerase chain reaction (RT-qPCR) analysis.

### Survival Rate, Rot Root Rate, Biomass, and Yield

In the field experiments, every 2 months during the whole experimental period (year 2016–2017 and year 2018–2019), the survival and root rot seedlings (see Supplementary Figure S1) of each plot were counted. At the harvest season, the plant biomass of each seedling and the yield of each plot were measured in November. Survival rate (%) = number of seedlings with health growth/number of transplanting seedlings × 100%; root rot rate (%) = number of root rot seedlings/number of transplanting seedlings × 100%.

In the pot culture experiment, Δ biomass [g/plant, fresh weight (FW)] = the weight of seedling at the harvest time (g/plant, FW) − the weight of seedling at the transplanting time (g/plant, FW).

### Soil TN, TK and AN, AK Determination

Soil samples collected from the field investigation, field experiment, and pot culture were air dried and smashed. Approximately 0.3 g soil was digested with 5 mL concentrated H_2_SO_4_ and 2 g catalyzer powder (mixed with 100 g K_2_SO_4_, 10 g CuSO_4_⋅5H_2_O, and 1 g Se), and the digestion solution was used for total nitrogen (TN) determination with the Kjeldahl method (Bremner, 1960). Approximately 0.3 g soil was digested with the NaOH-fusion method (Smith and Bain, 1982), and the digestion solution was used for TK determination with a flame photometer (PF6400, Shanghai, China). According to the Conway method (Bremner and Shaw, 1955), soil AN was incubated with 1.8 M NaOH and FeSO_4_ powder for 24 h. Soil AK was extracted with 1 M NH_4_AC according to Simard (1993) and determined with a flame photometer (PF6400, Shanghai, China).

### Plant N and K Determination

Plant samples collected from the field investigation, field experiments, and pot culture were air dried and smashed. Approximately 0.2 g (dry weight, DW) plant powder was digested with H_2_SO_4_–H_2_O_2_ according to Thomas et al. (1967). Then, the digestion was used for N content determination with the Kjeldahl method (Bremner, 1960) and for K content determination with a flame photometer (PF6400, Shanghai, China).

### Photosynthetic Index and Chlorophyll a + b (*Chl a* + *b*)

For the pot experiment, the photosynthetic parameters (*Pn*: net photosynthetic rate; *Gs*: stomatal conductance; *Ci*: intercellular CO_2_ concentration; *Ti*: transpiration rate) of leaves were measured with Li-6400XT portable photosynthesis system (Li-6400XT, Li-Cor, Inc., Lincoln, NE, United States). Then, leaves were collected for *Chl a* + *b* content determination according to Gitelson and Merzlyak (1997). Approximately 0.2 g FW leaf samples were extracted with 95% ethanol; the absorbance of extraction was measured at 665, 649, and 470 nm with a spectrophotometer (UV-2600, Shimadzu, Japan).

### Carbohydrate Content

For the pot experiment, seedling samples were collected in November 2017, divided into shoot and root, air dried, and smashed. Total soluble sugar (TSS) content was determined by anthrone method according to Siddiqi et al. (2002). Glucose (Glu) and sucrose (Suc) contents were measured by spectrophotometry method according to Stitt and Quick (1989).

### Saponin Content

All the root samples from the field investigation and field and pot experiments were air dried and smashed. Then, approximately 0.3 g DW root samples were extracted with methanol (MeOH) by sonication for 30 min, and saponin contents in the extraction were determined according to Xia et al. (2016). An Agilent 1200 series HPLC apparatus (high-performance liquid chromatography, Agilent, United States) and Shim-Pack PREP-ODS(H). Kit column (250 × 4.6 mm, 5 μm) was used for the determination. Chromatographic conditions were as follows: elution with 0–12 min, 18% acetonitrile, 12–35 min, 18–38% acetonitrile, 35–45 min, 38% acetonitrile, flow rate with 1.0 mL.min^–1^, injection volume with 20 μL, monitoring wavelength with 203 nm, and column temperature with room temperature.

### Gene Expression

For the hydroponic experiment, approximately 100 mg FW roots stored at −80°C were smashed to powder with liquid N_2_, and then RNA was extracted according to Livak and Schmittgen (2001). Samples were thawed at room temperature and homogenized in 1 mL of Trizol reagent (Invitrogen, Thermo Fisher Scientific, United States), and an RNA prep pure kit was used according to the manufacturer’s protocol. The quality of the RNA was determined using an ultraviolet spectrophotometer (Hoefer, Holliston, MA, United States), based on the ratio of the optical density at 260 nm to that at 280 nm (OD_260_/OD_280_), and was assessed by visual comparison of electrophoresis in a denaturing formaldehyde agarose gel with the 18s and 28s ribosomal RNAs.

For the reverse transcription, 4 μg of total RNA was mixed with 1 μL of Oligo (dT)_18_ primer (Thermo, Thermo Scientific Technology, United States) and 200 U of Revert Aid M-MuL Virus RT (Thermo) in the presence of 20 U of RiboLock RNase inhibitor (Thermo). Oligonucleotide primers for genes were designed using Primer Express Software (Applied Biosystems, Foster City, CA, United States). After reverse transcription, 20 ng of cDNA from the same cDNA batch was subjected to RT-qPCR to amplify all genes in triplicate in a total reaction volume of 15 μL using Roche SYBR Green Master mix (Roche) and the required amount of forward and reverse primers. Reactions were conducted on an LightCycler^®^ 96 (Roche, Roche Life Science, CH) using the following cycling conditions: preincubation at 95°C for 10 min, three-step amplification at 95°C for 10 s, 60°C for 30 s, and 72°C for 1 min. Expression of *PnACT2* was used as an internal control for target gene expression, and every gene primer sequence is shown in Supplementary Table S1. Every gene expression of each biological replication was determined with two technical replicates; in total, six values of every gene expression were gained. The gene expression was calculated based on the 2^–ΔΔCt^ method (Livak and Schmittgen, 2001).

### Statistical Analysis

All the data analyses were conducted with SPSS 18.0 (United States); Duncan test was used for one-way analysis of variance; linear and quadratic were used for curve fitting; Pearson correlation was used for correlation analysis.

## Results

### Investigation on the N/K Levels in Plants and Soils

**FIGURE 1:**
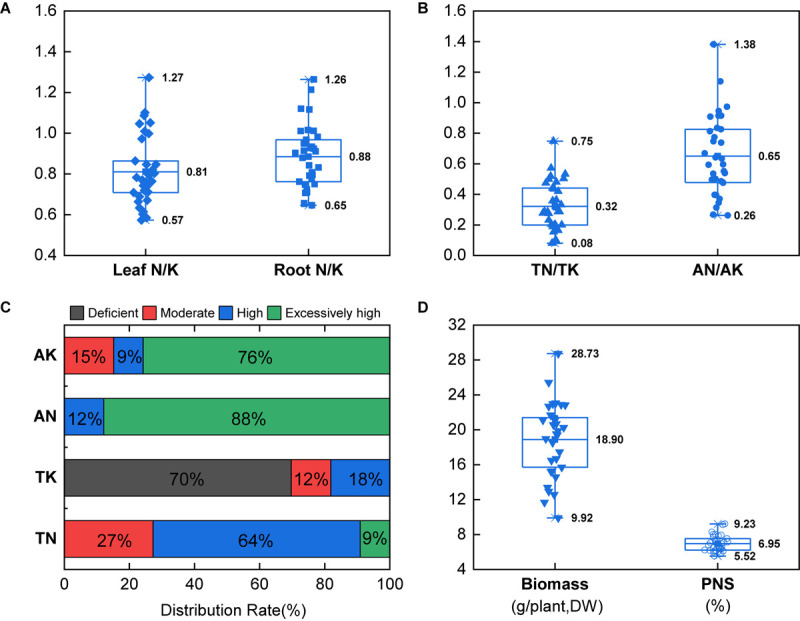
Field investigation of the N/K values in plants **(A)** and soils **(B)**, distribution of soil N and K **(C)**, and biomass and *P. notoginseng* saponins (PNS) **(D)**. The classification of soil nutrient was referred to the standard: TN < 1.0 g.kg^–1^ (deficient), 1.0 g.kg^–1^ ≤ TN < 1.0 g.kg^–1^ (moderate), 1.5 g.kg^–1^ ≤ TN < 2.0 g.kg^–1^ (high), TN ≥ 1.0 g.kg^–1^ (excessively high); AN < 90 mg.kg^–1^ (deficient), 90 mg.kg^–1^ ≤ AN < 120 mg.kg^–1^ (moderate), 120 mg.kg^–1^ ≤ AN < 150 mg.kg^–1^ (high), AN ≥ 150 mg.kg^–1^ (excessively high); TK < 15 g.kg^–1^ (deficient), 15 g.kg^–1^ ≤ TK < 20 g.kg^–1^ (moderate), 20 g.kg^–1^ ≤ TK < 25 g.kg^–1^ (high), TK ≥ 25 g.kg^–1^ (excessively high); AK < 100 mg.kg^–1^ (deficient), 100 mg.kg^–1^ ≤ AK < 150 mg.kg^–1^ (moderate), 150 mg.kg^–1^ ≤ AN < 200 mg.kg^–1^ (high), and AN ≥ 200 mg.kg^–1^ (excessively high). Boxplots center represents means; upper and lower represent 75 and 25% percentiles (*n* = 33). The error lines represent the maximum and minimum.

The N content in leaf of *P. notoginseng* was in the range of 15.21–26.08 g.kg^–1^, with an average of 19.42 g.kg^–1^; the N content in root was in the range of 4.85–10.80 g.kg^–1^, with an average of 7.63 g.kg^–1^. The K content in leaf of *P. notoginseng* was in the range of 15.36–33.84 g.kg^–1^, with an average of 24.69 g.kg^–1^; the K content in root was in the range of 5.65–12.73 g.kg^–1^, with an average of 8.74 g.kg^–1^ (Supplementary Table S2). The N/K value in leaf of *P. notoginseng* was in the range of 0.57–1.27, with an average of 0.81; the N/K value in root was in the range of 0.65–1.26, with an average of 0.88 (Figure 1A). The TN of the *P. notoginseng* cultivation soil was in the range of 1.13–3.62 g.kg^–1^, with an average of 2.09 g.kg^–1^; the AN was in the range of 97.29–397.76 mg.kg^–1^, with an average of 167.77 mg.kg^–1^; the TK was in the range of 3.20–16.70 g.kg^–1^, with an average of 8.23 g.kg^–1^; the AK was in the range of 111.70–627.65 mg.kg^–1^, with an average of 294.40 mg.kg^–1^ (Supplementary Table S2). The soil TN/TK value was in the range of 0.08–0.75, with an average of 0.32; the AN/AK value was in the range of 0.26–1.38, with an average of 0.65 (Figure 1B). In addition, 73% of the soil TN reached an excessively high level, and all of the soil AN reached an excessively high level (Figure 1C). However, 70% of the soil TK was at deficient level, whereas 85% of the soil AK reached an excessively high level (Figure 1C).

**FIGURE 2:**
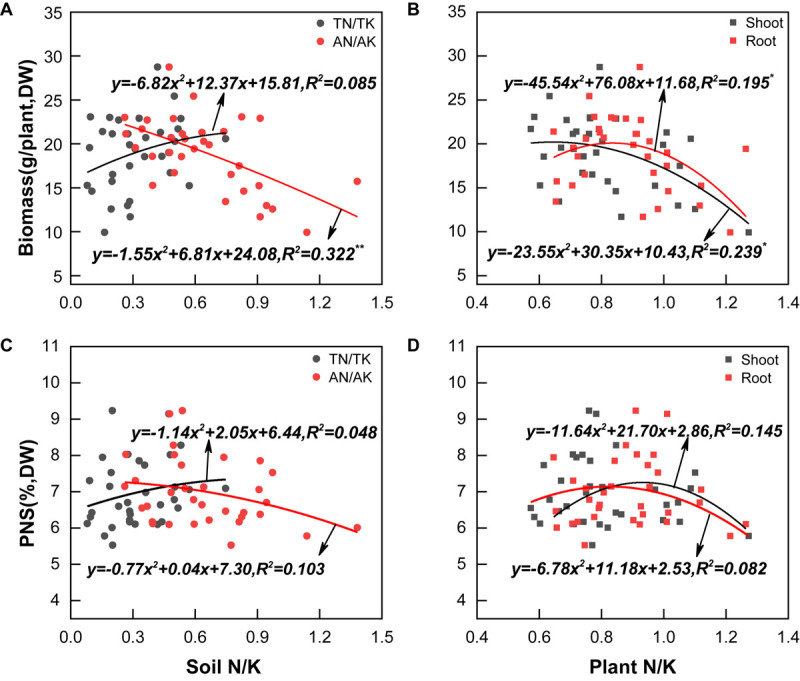
Correlation of the cultivation soil N/K values **(A,C)**, plants N/K values **(B,D)**, biomass levels **(A,B)**, and PNS **(C,D)**. All data are fitted with a linear regression, *n* = 33. mean significant * and ** correlation at *p* < 0.05 and *p* < 0.01, respectively.

The biomass of *P. notoginseng* was in the range of 9.92–28.73 g/plant, with an average of 18.90 g/plant; PNS in the main root was in the range of 5.52–9.23%, with an average of 6.95% (Figure 1D). Both the biomass and PNS increased at first and then became steady with the increase of soil TN/TK; the highest biomass and PNS content were reached when the soil TN/TK values were 0.91 and 0.90, respectively. However, biomass was significantly negatively correlated with the soil AN/AK value (*R*^2^ = 0.322^∗∗^, Figures 2A,C). Moreover, the biomass was significantly correlated with the N/K value of leaf or root (*R*^2^ = 0.239^∗^ and 0.195^∗^), which increased initially and then decreased. The highest biomass was reached when the N/K value of leaf and root was 0.64 and 0.84, respectively (Figure 2B). The PNS content also increased initially and then decreased with the increase of the N/K value of leaf or root; the highest PNS content was obtained when the N/K value of leaf and root was 0.93 and 0.82, respectively (Figure 2D). The above results indicated that *P. notoginseng* cultivation may cause an imbalance of soil N/K, which would significantly influence the growth and saponin contents.

### Response of Survival Rate to Different N and K Application

**FIGURE 3:**
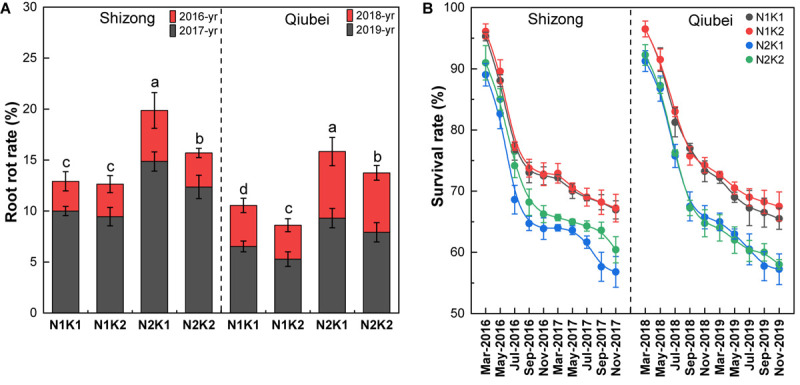
The root rot rate **(A)** and survival rate changing **(B)** of *P. notoginseng* seedlings under the conditions of different N and K application ratios. All data represent means ± SDs, *n* = 4; different small letters above the column represent significant difference among the four treatments at *p* < 0.05.

Root rot was mainly characterized by leaf chlorosis and root rot (Supplementary Figure S1), which is one of the highest mortality rates in *P. notoginseng* cultivation. Field experiments showed that the survival rates of the four treatments in Shizong and Qiubei were all gradually decreased with the growing time. At the harvest season, the N2K1 treatment had the highest root rot incidence and lowest survival rates among the four treatments both in Shizong and Qiubei. Compared with the N2K1 treatment, the root rot incidence of the N1K1, N1K2, and N2K2 treatments decreased by 34.9, 36.4, and 21.0% (Shizong), 33.3, 45.6, and 13.1% (Qiubei), respectively (Figure 3A). In comparison with the N2K1 treatment, the survival rates of the N1K1, N1K2, and N2K2 treatments increased by 17.8, 18.3, and 6.3% (Shizong) and 14.4, 17.9, and 1.3% (Qiubei), respectively (Figure 3B). Therefore, lowering N or increasing K conventional application level (N:K = 1:2) would be helpful to decrease the rot root rate and improve the survival rate of *P. notoginseng*.

### Response of Biomass and Yield to Different N and K Application

**FIGURE 4:**
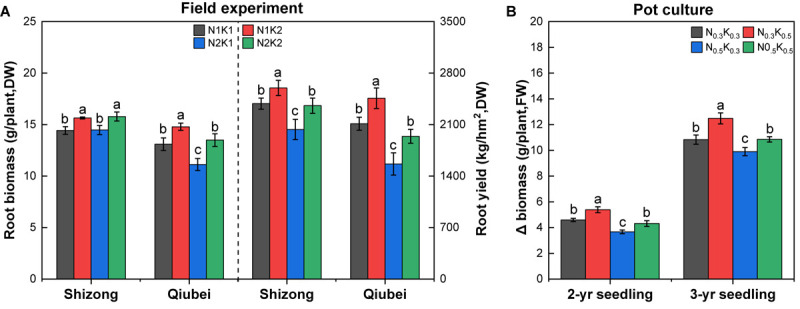
Root biomass and yield in the field experiments **(A)** and Δ biomass in the pot culture experiments **(B)** of *P. notoginseng* under the conditions of different N and K application ratios. All data represent means ± SDs, *n* = 4; different small letters above the column represent significant difference among the four treatments at *p* < 0.05.

Compared with the treatment of N2K1, except that the roots biomass of *P. notoginseng* under the N1K1 treatment at Shizong test site did not increase significantly, the N1K1 treatment at Qiubei test site increased by 17.7%; the N1K2 treatment increased by 5.7% (Shizong) and 32.9% (Qiubei); N2K2 treatment significantly increased by 9.6% (Shizong) and 21.3% (Qiubei). Compared with the N2K1 treatment, the root yield of *P. notoginseng* under N1K1 treatment was significantly increased by 17.3% (Shizong) and 35.0% (Qiubei); N1K2 treatment significantly increased by 27.8% (Shizong) and 57.1% (Qiubei); N2K2 treatment significantly increased by 15.9% (Shizong) and 24.1% (Qiubei) (Figure 4A).

Additionally, the pot culture experiments showed that the Δ biomass of 2- or 3-year seedlings treated with N_0_._5_K_0_._3_ was the lowest among the four treatments. In comparison with the Δ biomass of the N_0_._5_K_0_._3_ treatment, the N_0_._3_K_0_._3_ treatment significantly increased by 25.2% (2-year) and 9.4% (3-year); the N_0_._3_K_0_._5_ treatment significantly increased by 46.9% (2-year) and 26.1% (3-year); the N_0_._5_K_0_._5_ treatment significantly increased by 17.5% (2-year) and 9.7% (3-year) (Figure 4B). Briefly, lowering N or increasing K conventional application level (N:K = 1:2) would effectively increase the root biomass and yield of *P. notoginseng*.

### Response of PNS Content to Different N and K Application

**FIGURE 5:**
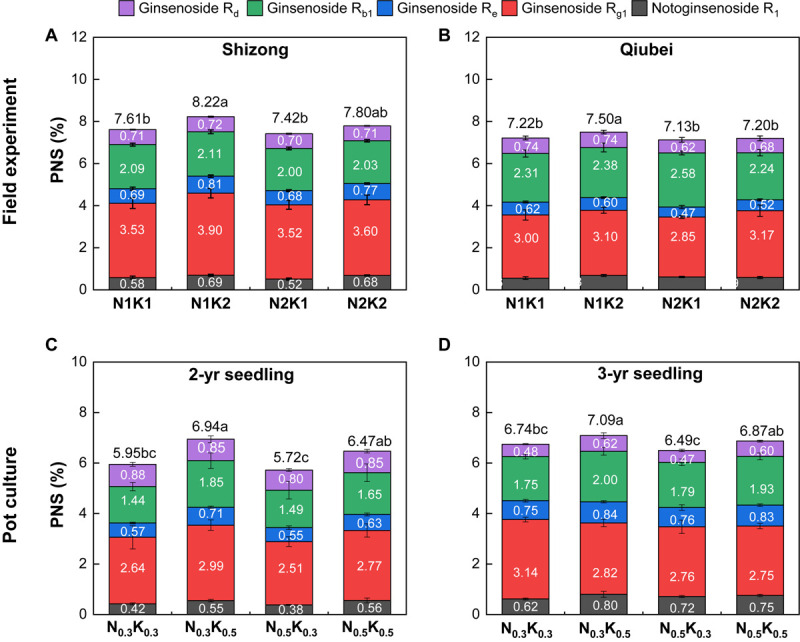
Root PNS content of *P. notoginseng* in the field experiment (**A:** Shizong; **B:** Qiubei) and in the pot culture experiments (**C:** 2-year seedling; **D:** 3-year seedling) under the conditions of different N and K application ratios. PNS, *Panax notoginseng* saponin. All data represent means ± SDs, *n* = 4; number in the box represents single saponin content; number above the box represents the sum of five single saponin content; different small letters above the column represent significant difference among the four treatments at *p* < 0.05.

Both the field and pot experiments showed that the PNS content in the roots of *P. notoginseng* under the treatment of N2K1 (or N_0_._5_K_0_._3_) was the lowest among the four treatments. Compared with the PNS content of the N2K1 treatment, only the N1K2 treatment significantly improved by 10% (Shizong, Figure 5A) and 5% (Qiubei, Figure 5B). Compared with the PNS content of the N_0_._5_K_0_._3_ treatment, the N_0_._3_K_0_._5_ treatment significantly improved by 21.3% (2 years, Figure 5C) and 9.1% (3 years, Figure 5D); the N_0_._5_K_0_._5_ treatment significantly improved by 11.5% (2 years, Figure 5C) and 5.5% (3 years, Figure 5D). Altogether, lowering N or increasing K conventional application level (N:K = 1:2) had a potential to improve the saponin accumulation of *P. notoginseng*.

### Correlation Between N/K Value and Biomass, and PNS Content

**FIGURE 6:**
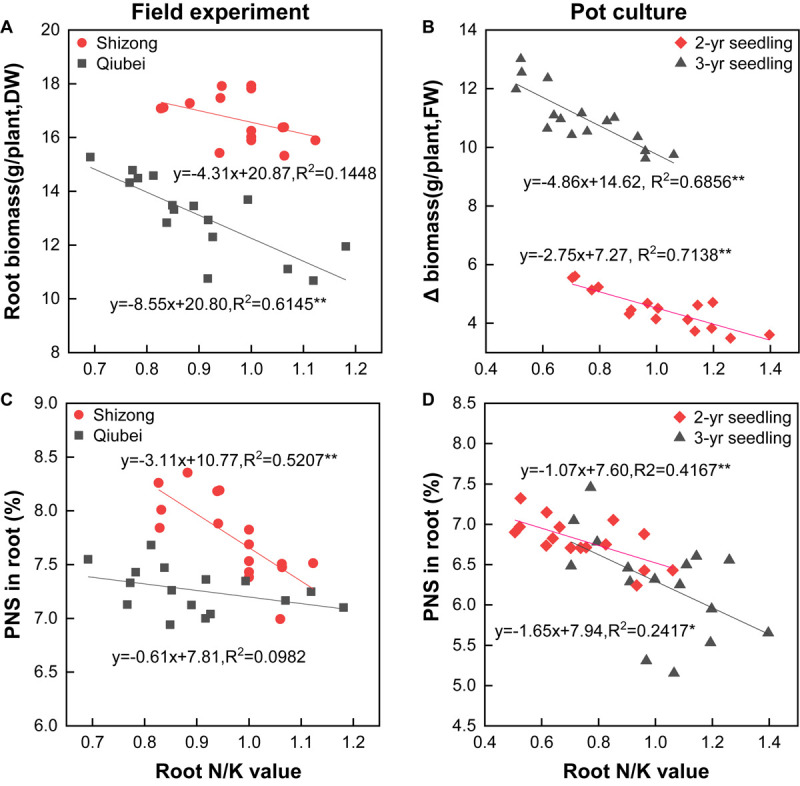
Correlations between N/K values and root biomass (**A**, field experiments; **B**, pot experiments), PNS content (**C**, field experiments; **D**, pot experiments) of *P. notoginseng* seedlings. All data are fitted with a linear regression, *n* = 16;* and ** denote significant correlation at *p* < 0.05 and *p* < 0.01, respectively.

The root biomass in the field and Δ biomass in the pot experiments all decreased with the increasing of N/K value, and the correlations of the field experiment in Qiubei and the pot culture experiments reached significantly levels (Figures 6A,B). Additionally, the PNS content in root significantly decreased with the increase of the N/K value both in the field and pot culture experiments, and all the correlations (except in the Qiubei field experiment) reached significant or highly significant levels (Figures 6C,D). The results suggest lowering N/K of *P. notoginseng* by lowering N and increasing K, which would promote the growth and saponin accumulation.

### Response of Photosynthesis and Carbohydrate to Different N and K Application

**FIGURE 7:**
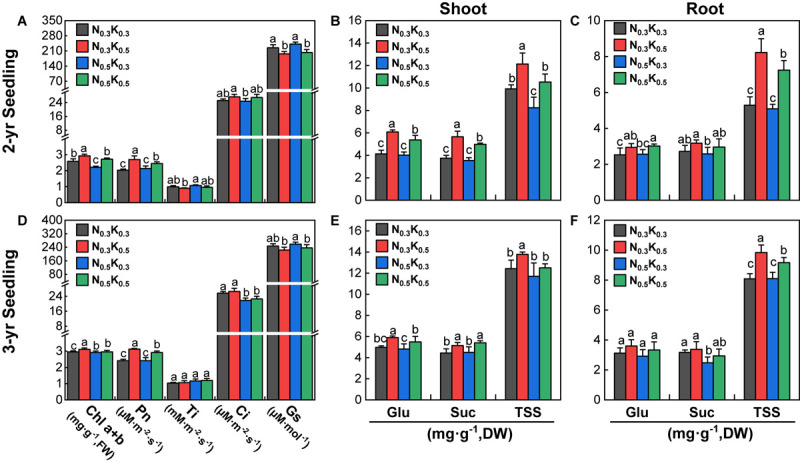
Photosynthetic **(A,D)** and sugar contents **(B,C,E,F)** of *P. notoginseng* seedlings in pot under the conditions of different N and K application ratios. *Chl a* + *b*, chlorophyll a + b; *Pn*, net photosynthesis rate; *Ti*, transpiration rate; *Ci*, intercellular CO_2_ concentration; *Gs*, stomatal conductance; Glu, glucose; Suc, sucrose; TSS, total soluble sugar. All data represent means ± SDs, *n* = 4; different small letters above the column represent significant difference among the four treatments at *p* < 0.05.

In the pot culture experiments, compared with N_0_._5_K_0_._3_ treatment, the *Chl a* + *b* content and *Pn* of 2- and 3-year seedlings were significantly improved under the N_0_._3_K_0_._5_ and N_0_._5_K_0_._5_ treatments (except the *Chl a* + *b* content of 3-year seedlings, Figures 7A,D). Moreover, the Suc, Glu, and TSS content in the shoot of 2- and 3-year seedlings (except TSS of 3-year seedlings) were significantly promoted by the N_0_._3_K_0_._5_ and N_0_._5_K_0_._5_ treatments (Figures 7B,E). The Glu, Suc, and TSS contents in the root of 2-year seedlings were significantly promoted by the N_0_._3_K_0_._5_ and N_0_._5_K_0_._5_ treatments, and the 3-year seedlings (expect Glu) were also significantly promoted by the N_0_._3_K_0_._5_ and N_0_._5_K_0_._5_ treatments (Figures 7C,F). Briefly, lowering N or increasing K application level (N:K = 1:2) would improve the carbohydrate accumulation of *P. notoginseng*.

### Correlations Between N/K or PNS and Photosynthetic Products, and Carbohydrate Contents

Contents of Chl a + b, *Pn*, and TSS were all significantly negatively correlated with the N/K values in shoot or root of 2- and 3-year seedlings and were all significantly positively correlated with the PNS contents in root of 2- and 3-year seedlings (except for the *Pn* of 3-year seedlings, Table 3). Thus, it implied that improving the carbohydrate accumulation can promote the saponins synthesis of *P. notoginseng*.

**TABLE 3:** Correlations between N/K values or PNS content and *Chl a* + *b*, *Pn*, and TSS content of *P. notoginseng* seedlings.

**Index**			**Shoot**			**Root**
			***Chl a* + *b***	***Pn***	**TSS**	**TSS**
2-year Seedling	Shoot	N/K	−0.845**	−0.586*	−0.762**	−0.739**
	Root	N/K	−0.870**	−0.605*	−0.745**	−0.774**
		PNS	0.666**	0.734**	0.672**	0.771**
3-year Seedling	Shoot	N/K	−0.551**	−0.564*	−0.647**	−0.560*
	Root	N/K	−0.542**	−0.549*	−0.643*	−0.540*
		PNS	0.585*	0.476	0.777**	0.643**

Note: *Chl a* + *b*, chlorophyll a + b; *Pn*, net photosynthesis rate; TSS, total soluble sugar. Correlation coefficients are calculated by Pearson correlation, *n* = 16. * and ** represent significant correlation at *p* < 0.05 and *p* < 0.01, respectively.

### Saponin Biosynthesis Pathway Gene Response to Different N/K Treatments

**FIGURE 8:**
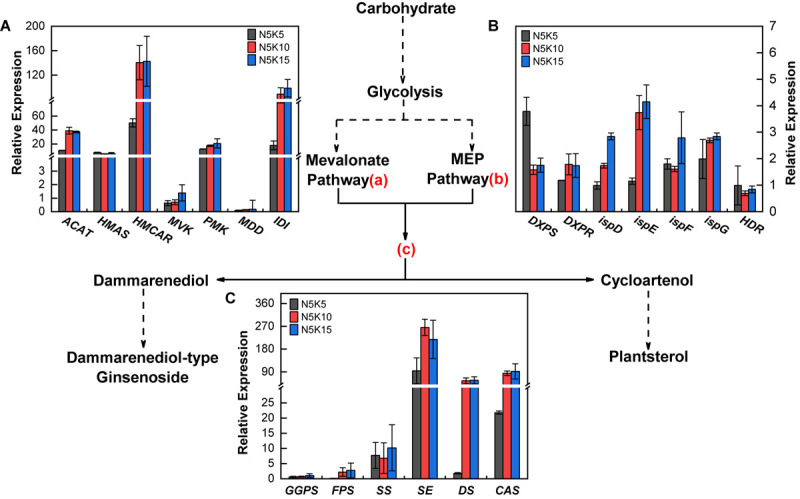
Expressions of saponin biosynthesis pathway genes in *P. notoginseng* response to 5 mM N with three K concentrations under hydroponic culture, respectively, were 5 mM (N5K5), 10 mM (N5K10), and 15 mM (N5K15). This pathway was referred to Liao et al. (2017). **(A)** Gene expression of the mevalonate pathway **(a)**; **(B)** gene expression of the MEP pathway **(b)**; **(C)** gene expression of the downstream pathway **(c)**. *ACAT*, acetyl-CoA acetyl transferase; *HMCAS*, 3-hydroxy-3-methylglutaryl coenzyme-A synthase; *HMCAR*, 3-hydroxy-3-methylglutaryl coenzyme-A reductase; *MVK*, mevalonate kinase; *PMK*, phosphomevalonate kinase; *MDD*, mevalonate diphosphate decarboxylase; *IDI*, isopentenyl diphosphate isomerase; *DXPS*, 1-deoxy-D-xylulose-5-phosphate synthase; *DXPR*, 1-deoxy-D-xylulose-5-phosphate reductoisomerase; *ispD*, 2-C-methyl-D-erythritol-4-phosphate cytidylyl-transferase; *ispE*, 4-diphosphocytidyl-2-C-methyl-D-erythritol kinase; *ispF*, 2-C-methyl-derythritol 2,4-cyclodiphosphate synthase; *ispG*, 4-hydroxy-3-methylbut-2-enyl-diphosphate synthase; *HDR*, 4-hydroxy-3-methylbut-2-enyl diphosphate reductase; *GGPS*, geranylgeranyl pyrophosphate synthase; *FPS*, farnesy pyrophosphate synthase; *SS*, squalene synthase; *SE*, squalene epoxidase; *DS*, dammarenediol-II synthase; *CAS*, cycloartenol synthase. All data represent means ± SDs, *n* = 4.

Compared to the N5K5 treatment (N:K = 1:1), both the treatments of N5K10 and N5K15 significantly upregulated the expression of *ACAT*, *HMCAR*, *IDI* in the mevalonate pathway (Figure 8A), *DXPR*, *ispD*, *ispE*, and *ispG* in the MEP pathway (Figure 8B), as well as *FPS*, *SE*, *DS*, and *CAS* (Figure 8C) of the PNS biosynthesis pathway. In addition, the differences of those gene expressions between the N5K10 and N5K15 treatments were not obvious. It suggests that increasing K application rate to N:K = 1:2 can upregulate 11 of 20 genes in the PNS biosynthesis pathway in *P. notoginseng*. Overall, it revealed that lowering N and increasing conventional application level (N:K = 1:2) improve the saponin content of *P. notoginseng*, which is mediated by upregulating the gene expression in the PNS biosynthesis pathway.

## Discussion

### Rational Application of N and K Can Reduce the Root Rot Rate in *P. notoginseng*

In *P. notoginseng* cultivation, root rot caused by fungal infection (Dong et al., 2016) is one of the most difficult diseases to control. Various studies have demonstrated that excessive application of N can increase the susceptibility of crops to pathogens (Veresoglou et al., 2013; Fagard et al., 2014). Wei et al. (2018) showed that compared to the normal application of N (225 kg.hm^–2^), excessive application of N (450 kg.hm^–2^) significantly increased the root rot incidence, due to the prevalence of certain soil fungi communities like *Fusarium* in *P. notoginseng* cultivation with high N levels. Similarly, this study showed that *P. notoginseng* with high-N had a significantly higher root rot rate and lower survival rate than that with low-N (Figures 3A,B). Moreover, root rot is also an important indication of continuous cropping obstacle, which also can be induced by imbalanced soil nutrients (Sun et al., 2015). Liu et al. (2014) have shown that the soil N/K value of continuous cropping *P. notoginseng* was higher than that of non-continuous. Soil N/K value was significantly increased when the N/K application rate increases from 1/1 to 1.6/1 (Wei et al., 2018), because of *P. notoginseng* demands more K than N (Ou et al., 2011), and K input is relatively lower than N input in *P. notoginseng* cultivation. Thus, it is inferred that rational application of N and K fertilizers can increase the resistance to pathogen and thus improve the resistance to root rot in *P. notoginseng* cultivation. The results of this study indicated that lowering the N and increasing the K application rate (N:K = 1:2) can decrease the N/K value of the cultivation soil (Supplementary Table S5) and increase the relative K content in *P. notoginseng* (Supplementary Table S3), which then leads to a reduction of the root rot rate (Figure 3B). That can be demonstrated by plants applied with abundant K can effectively resist with the infection of pathogens such as fungi and bacteria (Amtmann et al., 2008; Oosterhuis et al., 2013; Wang et al., 2013; Zörb et al., 2014), but the mechanism remains unclear and is an important direction for future studies.

### Rational Application of N and K Can Improve the Synergistic Effects on the Yield and Quality of *P. notoginseng*

Although the demand for N and K varies with crop species, rational fertilization of N and K can exert their synergistic effects on improving yield, such as keeping K application rate at 270 kg.hm^–2^, increasing N application rate from 225 to 450 kg.hm^–2^ (Wei et al., 2018), or keeping N application rate at 338 kg.hm^–2^, and increasing K application rate from 338 to 506 kg.hm^–2^ (Xia et al., 2016); the yield of *P. notoginseng* could be barely improved. Therefore, rational application levels of N and K with proper combined rates have a significant effect on yield improvement. However, little recommendation of rational N and K combined rate has been laid out in current literature. The present study showed that when lowering the N and increasing the K application rate (N:K = 1:2), *P. notoginseng* had the N/K value of 0.84 (Supplementary Table S3), which was consistent with the optimal N/K value obtained from the results of the investigation and non-linear curve fitting (Figure 2B). Furthermore, the root yield of *P. notoginseng* using the present fertilization method increased by 27.8–57.1% compared with that using the conventional fertilization approach (Figure 4A). Thus, a relatively balanced N and K application rate (N:K = 1:2) is suggested to fully exert their synergistic effects on the improvement of the yield of *P. notoginseng*.

Fertilization can affect secondary metabolites in plants by changing the nutrient status of their growing environment (Szakiel et al., 2011). The hypothesis of “C/N balance” and “growth/differentiation balance” suggests that plants growing in a nutrient-rich environment will allocate most of the nutrients for growth, rather than for carbon-based secondary metabolites synthesis; plants growing in a nutrient-deficient environment do inverse of that (Bryant et al., 1983; Herms and Mattson, 1992; Lerdau et al., 1994). As secondary metabolites are the quality indexes of medicinal plants, the trade-off between yield and quality should be considered in the fertilization of their cultivation. Application N improves the yield of *Centella asiatica* (Müller et al., 2013a,b) and *Stevia rebaudiana* (Barbet-Massin et al., 2015), but decreases their saponin contents. Similarly, PNSs are carbon-based secondary metabolites, which are the main quality ingredients of *P. notoginseng*. Our previous study also found that N fertilization improves the yield but decreases the PNS content (Ou et al., 2014, 2017). Interestingly, it has been reported that increasing the application of K fertilizers can improve the accumulation of PNS in *P. notoginseng* cultivation (Zheng et al., 2014; Xia et al., 2016). In view of that, even though N fertilization decreases the PNS content in *P. notoginseng* (Ou et al., 2014, 2017), PNSs also can be improved as long as the N/K application ratio is reasonable. This study showed that lowering the N and increasing the K application rate (N:K = 1:2) can promote PNS accumulation (Figure 5). The N/K value of *P. notoginseng* with this fertilization method was 0.84 (Supplementary Table S3), which was consistent with the optimal N/K value of 0.82 simulated with the survey results (Figure 2B). Thus, the synergistic effects of N and K will be improved at the application rate of N:K = 1:2, which could be beneficial for the growth and saponin accumulation of *P. notoginseng*.

### Rational Application of N and K Improves PNS Synthesis in *P. notoginseng*

Balanced fertilization can improve the *Pn* and carbohydrate accumulation in plants (Hu et al., 2015, 2017; Hou et al., 2018). The glycolysis of carbohydrates produces the substrates for the biosynthesis of PNS. Slight drought stress can enhance *Pn* and TSS content, thereby promoting the accumulation of PNS of *P. notoginseng* (Liao et al., 2017). However, excessive N application can inhibit photosynthesis in plants (Cao et al., 2009) and decrease the C/N value, thereby disturbing the secondary metabolism (Kong et al., 2017). Thus, it is inferred that the accumulation of PNS can also be promoted by a balanced fertilization. *Pn* in plants is mainly determined by stomatal conductance (*gs*), mesophyll conductance (*gm*), and net CO_2_ assimilation rate (*A*), as well as other parameters (Lu et al., 2016a,b), which in rice all increase with the decrease of the leaf N/K value (Hou et al., 2018). Accordingly, decreasing the N/K value in plants can enhance their *Pn*. Several studies have indicated that increasing the K application rate can enhance K uptake in cotton (Hu et al., 2015, 2017), decrease N uptake in wheat (Kong et al., 2014), and reduce the N/K value in rice (Hou et al., 2018). The results of the present study indicated that lowering the N and increasing the K application rate can decrease the N/K value of *P. notoginseng* and promote the PNS content (Figure 5 and Supplementary Tables S3, S4), as well as the *Pn* and TSS content (Figure 7). In addition, genes encoding enzymes involved in the biosynthesis pathway of PNS are regulated by environmental factors (Choi et al., 2005; Chen et al., 2007). For example, *DXS/CLA1*, *DXR*, and *GGPS* are related with saponin biosynthesis in *Medicago truncatula*, which are upregulated by low-N and low-P combined application (Bonneau et al., 2013). In order to explain the optimal application ratio of N and K fertilizers (1:2) obtained in field and pot experiments to promote the accumulation of PNS, we treated *P. notoginseng* with different application ratios of N and K fertilizers (1:1, 1:2, and 1:3) and studied the gene expression level in the saponin synthesis pathway. In this study, 11 of 20 genes of the PNS biosynthesis pathway were upregulated by increasing the K application rate to N:K = 1:2 (Figure 8). These findings were consistent with one of our previous studies, which showed that mild drought can enhance PNS accumulation by upregulating 18 genes of the biosynthesis pathway of PNS (Liao et al., 2017). In total, decreasing the N/K value of *P. notogingseng* by lowering the N and increasing the K application rate in its cultivation, that can improve Pn to accelerate carbohydrate accumulation, as well as upregulate the expression of genes related to saponin biosynthesis to promote the PNS accumulation.

## Conclusion

Lowering N and increasing K application rate (N:K = 1:2) can decrease root rot rate and increase the survival rate and biomass, as well as yield of *P. notoginseng*. Decreasing the N/K of *P. notoginseng* can improve its photosynthesis and sugar accumulation, as well as upregulate the expression of genes in the saponin biosynthesis pathway. Thus, the mechanism of lowering N and increasing K application rate (N:K = 1:2) enhances PNS accumulation and may relate to lower the N/K to improve the photosynthesis and to upregulate the gene expression to accelerate the PNS biosynthesis.

## Data Availability Statement

The original contributions presented in the study are included in the article/Supplementary Material. Further inquiries can be directed to the corresponding author/s.

## Author Contributions

DL and YY conceived and designed the experiments and provided funding. XO performed the experiments and analyzed the data. LG assisted in design experiment and data analysis. XC provided guidance in the field experiments. DZ guided the manuscript writing and revising. All authors contributed to the article and approved the submitted version.

## Conflict of Interest

The authors declare that the research was conducted in the absence of any commercial or financial relationships that could be construed as a potential conflict of interest.
