# Supplementary material for: Lowering Nitrogen and Increasing Potassium Application Level Can Improve the Yield and Quality of Panax notoginseng
Source: Front Plant Sci. 2020 Dec 21;11:595095. doi: 10.3389/fpls.2020.595095 (PMC7779559; doi:10.3389/fpls.2020.595095)
Supplement: Supplementary file 1 [file Data_Sheet_1.PDF]

Tab. S1 Primer sequences of genes in saponins biosynthesis pathway of *P.notoginseng*

Genes	5'-Forward-3'	5'-Reverse-3'
ACAT	TTCCATGCCACCAGCCACA	GGTCAAGCACCTGCAAGACAA
HMCAS	GTTGCGAAGTCCCAGTACGATGT	GGATGTTGACCTTCACGAAGACG
HMCAR	TTCTGGAAATTATTGCTCGGATAAG	AGGCTGCCACATTTGTCTTCAAC
MVK	TTGAATCTCCTGCTTCGGATGA	CCTCCACCACCAGCTCCTGT
PMK	ATGCTACTATGAAAATGGAAGGAGTC	TATGAACTGCCGAAATGCCAGA
IDI	TGTACCGAGAATCCGAGCTTATAGA	ATGCTCTCCCCACTTCCCATC
GGPS	ATCTATTGGGACAGAAGGGCTAGTA	AAATGGCTCCTAAAACTACAGATGC
GGR	ATGTTCTGAAGGAATTTCGAGTGTG	TCAAGAATATCATCCACCACCTGAA
SE	GACCCTTATGCGATCTCCATGACT	AATTCCTCCGAGGCTCAGATAATC
DS	TGACAACAGTGAAGCAGTTCGTAAG	CAAACATAAGACCTAGCATAGCCCA
CAS	ACAAGCATCAGATGGCTCATGGTA	CCAACCACCAGAAGCAAGTTGT
DXPS	GACCCTGGAGGACCTAACAT	TGGTCAGGTCTATGAGGCAAT
DXPR	GTCCTGCTCTTGTATGATTATGG	TCAATGTCTTTCACTTGCTCC
ispD	GATGACGATTCTTCAACTGGG	TCTTTGGCCTTTTCCTCCTG
ispE	TATTATTGCTGCGGGTTGTG	CGTCTTCAAGTGTCTTTGGTGTA
ispF	GAGTGAGCCTCGCAACCTTT	ACTCTGTTATGCCGCTACTTCTAT
ispG	AGGCTTTTGAGTCTCCATCCA	ATCGTGGAGACCTTGGTTCG
HDR	ACTTTCTTTTGGGCGACCTT	ACCGTTTGGTAGCCAGTTTT
ACT2	TCCAAGGGTGAATATGATGAATCG	AACCTCTCCAAAGAGAATTTCTGAGT

Tab. S2 Content of N and K in seedlings and soil of *P.notoginseng* from different cultivation regions

NO.	Site	Longitude	Latitude	N, K Content in *P. notoginseng* Seedlings						N,K Content in *P.notoginseng* Cultivation Soil						Whole Plant Biomass	Total Saponins Content in Root
				Leaf N (g/kg, DW)	Leaf K (g/kg, DW)	Leaf N/K Ratio	Root N (g/kg, DW)	Root K (g/kg, DW)	Root N/K Ratio	TN (g/kg, DW)	TK (g/kg, DW)	TN/TK Ratio	AN (mg/kg, DW)	AK (mg/kg, DW)	AN/AK Ratio	(g/plant, DW)	(%, DW)
1	Dongshan-Wenshan 1	104°14′47″	23°26′27″	15.33	26.24	0.58	6.54	8.42	0.78	1.13	11.71	0.10	97.29	117.90	0.83	23.07	6.30
2	Dongshan-Wenshan 2	104°19′24″	23°28′2″	18.50	27.60	0.67	5.69	8.02	0.71	1.49	16.25	0.09	111.30	355.58	0.31	19.54	7.30
3	Dongshan-Wenshan 3	104°19′24″	23°28′2″	17.05	28.27	0.60	5.85	8.28	0.71	1.25	15.51	0.08	129.11	326.28	0.40	15.26	6.12
4	Hongta-Yuxi	102°26′25″	24°25′25″	20.58	26.97	0.76	7.60	9.14	0.83	2.12	11.19	0.19	102.41	392.37	0.26	22.99	7.14
5	Jianshui-Honghe 1	102°42′20″	23°27′52″	16.00	27.92	0.57	7.59	9.59	0.79	1.89	3.65	0.52	149.89	435.90	0.34	21.69	6.55
6	Jianshui-Honghe2	102°59′30″	23°33′20″	21.73	21.53	1.01	6.51	8.19	0.79	1.60	3.69	0.43	397.76	627.65	0.63	21.28	6.64
7	Luxi-Honghe	103°54′24″	24°35′30″	17.54	25.48	0.69	8.22	8.87	0.93	1.60	6.89	0.23	139.50	376.90	0.37	22.69	6.60
8	Maguan-Wenshan 1	102°58′49″	25°11′8″	19.47	31.69	0.61	8.39	8.84	0.95	2.10	5.87	0.36	126.62	236.75	0.53	18.53	7.73
9	Maguan-Wenshan 2	104°5′11″	22°59′52″	17.87	17.08	1.05	8.89	7.97	1.12	3.62	13.16	0.27	130.59	138.27	0.94	12.95	6.70
10	Maguan-Wenshan 3	104°25'12"	22°52'48"	16.19	19.75	0.82	8.35	12.73	0.66	2.12	10.63	0.20	154.34	111.70	1.38	15.71	6.01
11	Maguan-Wenshan 4	104°25'12"	22°54'36"	19.35	17.58	1.10	8.65	8.81	0.98	3.01	15.30	0.20	155.82	160.10	0.97	12.58	7.52
12	Maguan-Wenshan 5	104°27′15″	22°57′3″	20.36	15.99	1.27	6.85	5.65	1.21	2.74	16.70	0.16	148.96	130.85	1.14	9.92	5.78
13	Maguan-Wenshan 6	104°0′50″	22°53′36″	15.21	17.96	0.85	7.04	6.93	1.02	1.54	15.09	0.10	152.85	183.11	0.83	14.59	6.42
14	Mengzi-Honghe	103°12′23″	23°12′23″	17.49	22.91	0.76	7.32	8.69	0.84	2.51	7.60	0.33	225.57	246.81	0.91	22.89	7.85
15	Mile-Honghe	103°15′0″	24°26′46″	20.18	20.76	0.97	7.20	6.43	1.12	2.27	3.97	0.57	158.27	174.22	0.91	15.22	7.05
16	Pingba-Wenshan	104°8′6″	23°15′18″	16.35	20.87	0.78	6.98	6.90	1.01	2.00	4.22	0.47	138.72	290.57	0.48	18.98	9.14
17	Qilin-Qujing 1	104°3′43″	25°10′29″	20.85	24.63	0.85	8.43	9.60	0.88	2.22	4.18	0.53	176.60	356.21	0.50	22.86	8.28
18	Qilin-Qujing 2	104°3′48″	25°10′23″	18.95	25.65	0.74	10.16	10.49	0.97	2.02	4.20	0.48	155.82	311.32	0.50	16.69	8.02
19	Qiubei-Wenshan 1	104°8′3″	24°9′34″	19.41	29.25	0.66	5.01	7.67	0.65	1.73	6.09	0.28	217.31	290.85	0.75	13.40	6.46
20	Qiubei-Wenshan 2	104°10′32″	23°59′23″	19.41	26.98	0.72	5.63	8.72	0.65	2.34	15.15	0.15	186.58	252.75	0.74	21.40	7.94
21	Shilin-Kunming	103°64′72″	24°74′78″	18.04	25.35	0.71	7.62	6.03	1.26	1.94	6.24	0.31	138.72	232.85	0.60	19.41	6.10
22	Shiping-Honghe	102°34′16″	23°27′9″	16.69	15.36	1.09	5.01	6.69	0.75	3.01	4.02	0.75	231.82	420.67	0.55	20.58	7.09

23	Shizong-Qujing	104°0′49″	24°53′50″	21.49	28.94	0.74	6.80	9.40	0.72	2.30	5.23	0.44	143.37	363.27	0.39	18.56	6.17
24	Songming-Kunming	102°51′31″	25°14′11″	20.05	26.36	0.76	10.80	11.86	0.91	1.55	7.68	0.20	226.14	420.17	0.54	21.13	9.23
25	Xiaoshao-Kunming	104°37′32″	23°25′32″	20.04	26.67	0.75	8.05	9.97	0.81	2.42	6.68	0.36	202.96	418.42	0.49	20.70	6.97
26	Xichou-Wenshan 1	104°23′40″	23°3′25″	19.32	24.32	0.79	6.79	7.35	0.92	2.70	6.43	0.42	157.34	331.57	0.47	28.73	6.09
27	Xichou-Wenshan 2	104°38′14″	23°23′26″	20.79	29.34	0.71	10.66	11.67	0.91	2.06	7.44	0.28	155.82	583.47	0.27	21.11	8.03
28	Xundian-Kunming	103°20′32″	25°44′28″	18.08	28.56	0.63	4.85	6.37	0.76	2.16	4.32	0.50	183.87	310.32	0.59	25.42	6.78
29	Yanshan-Wenshan 1	104°18′12″	23°39′32″	22.73	28.32	0.80	10.12	10.60	0.95	1.61	3.20	0.50	157.30	245.28	0.64	20.27	7.13
30	Yanshan-Wenshan 2	104°18′12″	23°39′32″	24.97	28.91	0.86	10.25	10.99	0.93	2.78	9.69	0.29	210.32	230.02	0.91	11.69	6.37
31	Yanshan-Wenshan 3	104°26′17″	23°42′27″	26.08	33.84	0.77	8.18	10.96	0.75	1.35	6.68	0.20	186.58	241.10	0.77	16.50	5.52
32	Yiliang-Kunming	103°4′28″	24°34′52″	23.37	23.42	1.00	7.32	8.10	0.90	2.22	7.80	0.29	184.34	275.97	0.67	19.89	6.21
33	Zhaokua-Qujing	103°44′8″	24°25′3″	21.45	20.40	1.05	8.52	8.44	1.01	1.44	5.05	0.29	102.40	125.95	0.81	17.47	6.17
	Minimum			15.21	15.36	0.57	4.85	5.65	0.65	1.13	3.20	0.08	97.29	111.70	0.26	9.92	5.52
	Maximum			26.08	33.84	1.27	10.80	12.73	1.26	3.62	16.70	0.75	397.76	627.65	1.38	28.73	9.23
	Average			19.42	24.69	0.81	7.63	8.74	0.88	2.09	8.23	0.32	167.77	294.40	0.65	18.90	6.95

Note: The investigation was conducted from 2013 to 2014. Soil of 0~20 cm layer from each culitivation region was collected using "five-point" method, then mixed, air dried, grounded for N, K content analysis. 20 plants of 3-yr old *P.notoginseng* seedlings from each cultivation region were randomly sampled, then washed off, dried, grounded for N, K and saponins content analysis. TN: total nitrogen; TK: total potassium; AN: available nitrogen; AK: available potassium.

Tab. S3 Content of N and K in *P.notoginseng* responses to balanced N and K application under field condition

Treatment		Shoot			Root		
		N ($mg·kg^{-1}$, DW)	K ($mg·kg^{-1}$, DW)	N/K	N ($mg·kg^{-1}$, DW)	K ($mg·kg^{-1}$, DW)	N/K
**Shizong**	N1K1	28.96±0.63b	31.35±0.6b	0.92±0.02b	16.03±0.53b	16.03±0.31b	1.00±0.05b
	N1K2	27.62±0.74c	34.13±1.29a	0.81±0.04c	14.69±0.32c	17.44±0.36a	0.84±0.03c
	N2K1	30.80±0.43a	30.55±0.33b	1.01±0.02a	17.18±0.92a	16.18±0.33b	1.06±0.05a
	N2K2	29.36±0.21b	33.10±0.97a	0.89±0.03b	16.46±0.54ab	16.96±0.74a	0.97±0.03b
**Qiubei**	N1K1	23.16±1.09b	28.89±1.15c	0.80±0.07bc	15.09±0.88a	16.20±0.83a	0.93±0.04b
	N1K2	24.50±1.80b	33.36±1.98a	0.73±0.02c	12.63±0.52b	16.56±1.11a	0.76±0.05c
	N2K1	27.11±1.16a	30.10±1.29bc	0.90±0.06a	15.34±0.90a	14.40±1.33b	1.07±0.11a
	N2K2	26.64±0.86a	32.34±1.97ab	0.83±0.06ab	13.02±0.38b	15.77±0.51ab	0.83±0.04bc

Note: Mean ± SD., n=4, small letters in the same column mean significant difference at $p < 0.05$.

Tab. S4 Content of N and K in *P.notoginseng* responses to balanced N and K appliication under pot culture condition

Treatment		Shoot			Root		
		N ($g \cdot kg^{-1}$, DW)	K ($g \cdot kg^{-1}$, DW)	N/K	N ($g \cdot kg^{-1}$, DW)	K ($g \cdot kg^{-1}$, DW)	N/K
**2-yr seedling**	$N_{0.3}K_{0.5}$	24.16±0.99b	24.68±1.39c	0.98±0.08b	14.98±0.80c	14.80±1.43b	1.02±0.12b
	$N_{0.3}K_{0.5}$	22.78±1.08b	29.25±1.30a	0.78±0.04c	13.92±0.41c	18.69±0.90a	0.74±0.04c
	$N_{0.3}K_{0.5}$	25.88±0.76a	22.60±1.40d	1.15±0.10a	16.53±0.64b	13.32±0.83b	1.25±0.11a
	$N_{0.3}K_{0.5}$	27.42±1.21a	27.19±0.71b	1.01±0.07b	17.96±1.24a	17.35±0.76a	1.04±0.11b
**3-yr seedling**	$N_{0.3}K_{0.5}$	26.10±0.63b	34.03±2.25b	0.77±0.05b	15.49±0.61b	23.13±2.16b	0.67±0.06c
	$N_{0.3}K_{0.5}$	25.32±0.91b	38.29±1.11a	0.66±0.04c	14.74±0.87b	27.23±1.07a	0.54±0.05d
	$N_{0.3}K_{0.5}$	30.83±1.26a	31.29±0.99c	0.99±0.04a	20.05±1.21a	20.49±0.95c	0.98±0.06a
	$N_{0.3}K_{0.5}$	29.94±1.64a	35.85±1.49b	0.84±0.06b	19.18±1.58a	24.87±1.43b	0.77±0.08b

Note: Mean ± SD., n=4, small letters in the same column mean significant difference at $p < 0.05$.

Tab. S5 Content of N and K in soil responses to balanced N and K application under field condition

Treatment		TN (g/kg)	TK (g/kg)	TN/TK	AN (mg/kg)	AK (mg/kg)	AN/AK
**Shizong**	N1K1	1.24±0.07b	7.76±0.37b	0.16±0.02c	152.4±12.2b	167.0±38.0b	0.96±0.30ab
	N1K2	1.21±0.07b	9.66±0.43a	0.13±0.02d	158.2±11.7b	236.2±17.2ab	0.67±0.06b
	N2K1	2.23±0.09a	8.06±0.74b	0.28±0.06a	191.7±4.6a	164.3±46.4b	1.23±0.30a
	N2K2	2.17±0.10a	9.16±0.88a	0.24±0.04b	184.2±8.6a	217.1±28.8a	0.86±0.13b
**Qiubei**	N1K1	1.24±0.07b	7.76±0.37b	0.16±0.02c	152.4±12.2b	167.0±38.0b	0.96±0.30ab
	N1K2	1.21±0.07b	9.66±0.43a	0.13±0.02d	158.2±11.7b	236.2±17.2ab	0.67±0.06b
	N2K1	2.23±0.09a	8.06±0.74b	0.28±0.06a	191.7±4.6a	164.3±46.4b	1.23±0.30a
	N2K2	2.17±0.10a	9.16±0.88a	0.24±0.04b	184.2±8.6a	217.1±28.8a	0.86±0.13b

Note: Mean ± SD., n=4, small letters in the same column mean significant difference at $p < 0.05$.TN: total nitrogen; TK: total potassium; AN: available nitrogen; AK: available potassium.

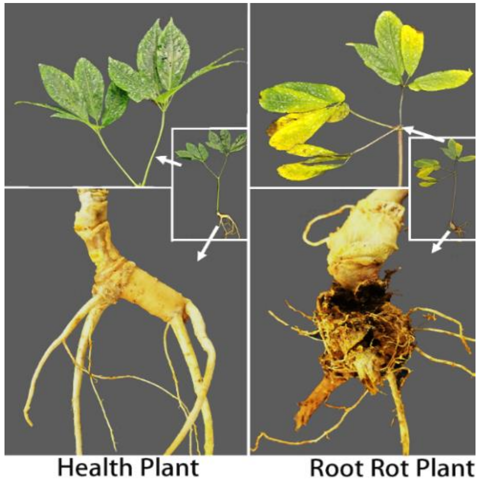

Fig. S1 Apparent of health and root rot plant of *P. notoginseng* seedlings
